# Sex differences in the effect of viable cell-containing and heat-killed *Roseburia faecis* on repeated water avoidance stress-induced irritable bowel syndrome Wistar rat model

**DOI:** 10.1371/journal.pone.0360294

**Published:** 2026-11-13

**Authors:** Sungchan Ha, Nayoung Kim, Ryoung Hee Nam, Isom Jin, Eun Hye Kim, Chin-Hee Song, Byoung-Kook Kim, Sang-Hwa Choi, Kisung Kang, Wonseok Lee, Dong Ho Lee

**Affiliations:** 1 Department of Health Science and Technology, Graduate School of Convergence Science and Technology, Seoul National University, Seoul, South Korea; 2 Department of Internal Medicine, Seoul National University Bundang Hospital, Seongnam, South Korea; 3 Research Center for Sex- and Gender-Specific Medicine, Seoul National University Bundang Hospital, Seongnam, South Korea; 4 Department of Internal Medicine and Liver Research Institute, Seoul National University College of Medicine, Seoul, South Korea; 5 Central Research Institute, MEDIOGEN Co., Ltd., Chungju, South Korea; 6 R&D Center, Bio Bank Healing, Seongnam, South Korea; Wuhan Polytechnic University, CHINA

## Abstract

**Background:**

*Roseburia faecis* has been reported to mitigate irritable bowel syndrome (IBS), which is characterized by altered intestinal motility, mucosal immune activation, and gut microbial dysbiosis. This study aimed to evaluate the sex-dependent effects of heat-killed *R. faecis* (HK-BBH024) and a viable cell-containing *R. faecis* preparation (L-BBH024) in a repeated water avoidance stress (rWAS)-induced IBS Wistar rat model.

**Methods:**

Fecal pellet output (FPO) and colonic mast cell infiltration were measured in the rWAS IBS model induced in 7-week Wistar rats (males and females). Gut microbial composition and function were analyzed by 16S rRNA sequencing and Kyoto Encyclopedia of Genes and Genomes pathway prediction, with or without BBH024 treatment.

**Results:**

The marked rWAS-induced increase in FPO in both sexes was reduced by HK-BBH024 and L-BBH024. rWAS also increased colonic mast cell infiltration in the proximal colon and the mRNA expression of protease-related genes, whereas L-BBH024 attenuated these sex differences. Specifically, L-BBH024 was associated with a borderline decrease in mast cell infiltration and suppressed *Prss1* and *Prss2* mRNA expression in females, whereas *Prss3* expression was reduced in males. Microbiota profiling showed that rWAS-associated alterations in beta diversity, selected bacterial taxa, linear discriminant analysis-derived microbial biomarkers, and predicted KEGG functional pathways were more evident in females.

**Conclusions:**

Both preparations reduced rWAS-induced FPO in both sexes. In the L-BBH024 experiment, additional changes in mast cell infiltration, protease-related gene expression, and microbial features were more evident in females.

## Introduction

Irritable bowel syndrome (IBS) is a prevalent disorder of the gut-brain interaction (DGBI) characterized by recurrent abdominal pain and altered bowel habits without identifiable structural abnormalities [1]. The global prevalence of IBS is estimated to be approximately 11%, although estimates vary considerably according to the geographic region, diagnostic criteria, and study methodology [2]. IBS has a multifactorial pathophysiology involving psychological stress, altered intestinal permeability, mucosal immune activation, gut microbial dysbiosis, abnormal motility, and visceral hypersensitivity [3]. Psychological stress is particularly important in the onset and exacerbation of IBS symptoms, partly through its effects on gut motility, mucosal barrier function, neuroimmune signaling, and visceral sensitivity [4,5]. Repeated water avoidance stress (rWAS)-induced IBS in animals is widely recognized model that recapitulates stress-associated IBS pathophysiology and has been reported to induce increased fecal pellet output (FPO), colonic mucosal mast cell infiltration, mucosal inflammatory responses, and gut microbial dysbiosis [6,7]. Probiotics have been investigated as potential therapeutic approaches to restore gut microbial balance, enhance intestinal barrier integrity, and attenuate intestinal inflammation [8,9]. Among the commensal gut bacteria, members of the genus *Roseburia* are butyrate-producing taxa that may contribute to mucosal barrier maintenance and immune homeostasis [10]. In an rWAS-induced IBS rat model, *R. faecis* administration reduced WAS-induced increases in FPO and partially attenuated colonic mucosal mast cell infiltration [6]. The findings suggest that *R. faecis* modulates stress-induced IBS-like responses and intestinal mucosal microinflammation. Although probiotic research has traditionally focused on live microorganisms, it remains unclear whether microbial viability is essential for physiological effects. The “probiotic paradox” suggests that both live and dead microbial cells can act as biological response modifiers, with live cells affecting gut microbial composition and immune responses, while non-viable cell components probably inducing anti-inflammatory responses in the gastrointestinal tract [11]. In line with this concept, postbiotics, defined as preparations of inanimate microorganisms and/or their components that confer health benefits to the host, have gained attention for their potential to modulate host immunity and intestinal barrier function, while offering advantages in safety, storage stability, and standardization [12,13]. In Japan, inactivated lactic acid bacteria and post-biotic-like preparations have been actively investigated and used in functional food products [14–16]. These non-viable microbial preparations may exert their effects through heat-stable or released bacterial components, metabolites, and secreted factors that support barrier integrity and attenuate inflammation [17,18]. Notably, heat inactivation does not necessarily abolish microbial bioactivity, as pasteurized *Akkermansia muciniphila* showed greater metabolic benefits than live bacteria in a high-fat diet-induced metabolic disorder model, suggesting a role for heat-stable microbial components in host-response modulation [19]. As IBS is more prevalent in women and gut microbiota exhibits sex-specific differences, probiotics may exert sex-specific therapeutic effects [7,20]. Additionally, sex hormones is well known to affect the gut microbiome, particularly through immune activity [21]. Furthermore, both estrogen and androgens are affected by gut microbiome, especially by β-glucuronidase-producing microbiome in the menopause and in old males [22]. Thus, there are biologically plausible mechanisms linking sex differences to gut microbiota composition, immune signaling, and visceral sensitivity [21–23]. Finally, we hypothesized that heat-killed *R. faecis* (HK-BBH024) and a viable-cell-containing *R. faecis* preparation (L-BBH024) could reduce IBS-related responses, with potentially different response patterns between male and female rats. Accordingly, this study aimed to evaluate the effects of HK-BBH024 and L-BBH024 with a focus on sex differences in a rWAS-induced IBS Wistar rat model.

## Methods

### Preparation and characterization of *R. faecis* BBH024 preparations

The BBH024 strain of *R. faecis* used in our previous study was supplied by Mediogen Co., Ltd. (Korea) in two forms: a heat-killed preparation and a viable cell-containing preparation, hereafter referred to as HK-BBH024 and L-BBH024, respectively. For the heat-killed preparation, BBH024 was autoclaved at 121°C for 30 min, and the L-BBH024 preparation was processed without thermal treatment. The bacterial density was determined using a hemocytometer combined with fluorescence microscopy. A LIVE/DEAD^®^ BacLight™ Bacterial Viability Kit (L7012; Thermo Fisher Scientific, Waltham, MA, USA) was used to differentiate between viable and non-viable cells. Viable cells with intact membranes were stained green with SYTO 9, while nonviable cells with damaged membranes were stained red with propidium iodide. Viable and nonviable cell populations were quantified as active fluorescent units (AFU) and non-active fluorescent units (n-AFUs), respectively. The heat-killed preparation contained 5.8 × 10^11^ cells/g. The L-BBH024 preparation contained a total of 1.00 × 10^11^ cells/g, consisting of 5.00 × 10^9^ AFU/g and 9.50 × 10^10^ n-AFU/g. Thus, AFU accounted for approximately 5% of the total fluorescently enumerated cells based on LIVE/DEAD membrane-integrity staining. The relatively low viability of the L-BBH024 preparation may reflect the susceptibility of this obligate anaerobic strain to oxygen exposure during downstream processing and formulation.

### Animals and water stress protocol

Six-week-old male and female Wistar rats (Orient Co., Ltd.) were housed in cages (two animals per cage) at 23°C under a 12-hour light/dark cycle. The animals were maintained under specific pathogen-free conditions, and all procedures were conducted in accordance with the guidelines of the Institutional Animal Care and Use Committee of Seoul National University Bundang Hospital (approval no. BA-2112-333-002-06). This study was not designed as a survival study, and death was not used as an experimental endpoint. Following a 1-week acclimation period, the rats were randomly assigned to three experimental groups (n = 6 per group): control (CON), water avoidance stress (WAS), and WAS with bacterial administration (WAS + bacteria). The sample size of six animals per group was selected based on our previous experience with the rWAS rat model [6] and was considered to be enough for this exploratory study while minimizing the number of animals used. No formal a priori power calculation was performed. Experiments were conducted in two separate rounds: the CON, WAS, and WAS + HK-BBH024 groups, and subsequently, the CON, WAS, and WAS + L-BBH024 groups. Each experimental round included its own independent CON and WAS groups, and HK-BBH024 and L-BBH024 were therefore not directly compared between experimental rounds. BBH024 preparations were suspended in 400 μL of sterile PBS and administered once daily by oral gavage at a dose of 10 mg per rat per day beginning 3 days before the first WAS session and continuing throughout the 10-day WAS protocol. The HK-BBH024 and L-BBH024 preparations were administered at the same preparation mass and were not matched by total or viable cell number. Based on the measured cell densities, the daily doses corresponded to 5.8 × 10^9^ total cells for HK-BBH024 and 1.0 × 10^9^ total cells for L-BBH024 per rat; the latter included approximately 5.0 × 10^7^ AFU and 9.5 × 10^8^ n-AFU per rat. The WAS groups received 400 μL of sterile PBS by oral gavage according to the same schedule, whereas the CON groups received no administration. The rWAS protocol was performed as previously reported [6,7]. To account for sex-related physiological differences, male and female rats were placed on glass platforms of different dimensions (5.8 × 5.8 × 6.0 cm for males; 5.0 × 5.0 × 6.0 cm for females) positioned at the center of standard plastic cages (26.7 × 48.3 × 20.3 cm) containing water up to 1 cm below the platform. Rats were subjected to WAS for 1 hour daily between 08:00 and 10:00 for 10 consecutive days. Control animals were placed on the same platform in dry cages for equivalent periods. The rats were housed individually during each session, and stress-induced defecation was assessed by counting the total number of fecal pellets expelled at the end of each session. Female FPO values were adjusted by multiplying the raw pellet counts by the ratio of male to female body weight, as previously described [6]. Body weight normalization of FPO has also been used in our previous rWAS studies to account for sex-related differences in body weight [5,24]. To assess whether this adjustment affected comparisons between sexes, raw, unadjusted FPO values were additionally analyzed and are presented in S1 Fig. After each session, the animals were returned to their home cages.

Animal health and welfare were checked daily throughout the experimental period and additionally before and after each WAS or dry-platform session by trained personnel. These checks were based on routine visual inspection of general appearance, posture, activity, grooming condition, respiration, access to food and water, and signs of pain or distress. Body weight was recorded during the experimental period, and body weight changes are presented in S2 Fig A and B. Predefined humane endpoint criteria, in accordance with the approved IACUC protocol, included body weight loss exceeding 20% from baseline, epistaxis, or other abnormal clinical signs indicating severe distress or poor health. Animals showing these signs were to be immediately removed from the experiment and humanely euthanized following consultation with veterinary staff. No animals died or met humane endpoint criteria before the scheduled endpoint.

Following completion of the WAS protocol in each round, all animals were euthanized at the scheduled endpoint on day 11 for tissue collection. Rats were euthanized via gradual CO_2_ displacement (1.5–2.0 L/min) in a dedicated chamber, and death was confirmed by the absence of heartbeat before tissue collection. Colon mucosa and fecal samples were collected and stored at −80℃. No analgesics or anesthetics were administered during the WAS protocol because no invasive or surgical procedures were performed before the scheduled euthanasia, and such interventions could affect stress-related physiological responses. All procedures, including animal handling, rWAS exposure, monitoring, bacterial administration, and euthanasia, were performed by personnel trained in laboratory animal care and handling. All efforts were made to minimize animal suffering and distress.

### Colonic mucosal mast cell assessment

For histological assessment of mast cell infiltration, a 1-cm segment of the proximal colon was collected. The tissue was fixed in 10% buffered formalin, embedded in paraffin, and sectioned at a thickness of 4-μm. To identify mucosal mast cells, the sections were stained with toluidine blue, which stains mast cell granules purple. The number of positive cells was quantified relative to the total analyzed mucosal surface area and reported as mast cells per m㎡ of colonic mucosa following established protocols [6,7].

### Real-time quantitative PCR

Total RNA was extracted from the colonic mucosa using TRIzol reagent, and the concentration was verified using a NanoDrop 1000. After cDNA synthesis using a High-Capacity Reverse Transcription Kit, RT-qPCR was performed using an ABI Viia7 system with SYBR Green I Master Mix. GAPDH (GAPDH-F: 5'-GGGCTGGCATTGCTCAATG-3'; GAPDH-R: 5'-AGGGAGATGCTCAGTGTTGGG-3') as the internal reference for normalization. Target genes and primer sequences were as follows: *PRSS1* (F: 5'-CCAAGTGAGACTGGGAGAGC-3', R: 5'-GTTGGGGTGCTTGATGATCT-3'), *PRSS2* (F: 5'-CCAAGTGAGACTGGGAGAGC-3', R: 5'-TCCTATCGAAGTTGGGATGC-3'), *PRSS3* (F: 5'-GGAGTTGCTGTTGCTTTCCCT-3', R: 5'-TCTCACTTGGATGCGGGTCT-3'), and IL-1β (F: 5'-GCATCCAGCTTCAAATCTCA-3', R: 5'-ATCATCCCACGAGTCACAGA-3').

### Fecal Microbiota analysis

Bacterial genomic DNA was isolated from frozen fecal samples using the QIAamp DNA Stool Mini Kit (Qiagen, Hilden, Germany). Sequencing of the V3–V4 regions was conducted at CJ Bioscience, Inc [25,26].. PCR amplicons were purified and sequenced by CJ Bioscience, Inc. (Seoul, South Korea). Following primer trimming and quality filtering (Hidden Markov model/proprietary tools), the paired-end reads were merged. Sequence data processing was conducted using a proprietary bioinformatics workflow at CJ Bioscience following established protocols [25,26]. Paired-end reads were merged and low-quality, chimeric, or non-target sequences were filtered out. Taxonomic assignments were performed against the EzBioCloud 16S rRNA database using USEARCH software (ver. 8.1.1861) and operational taxonomic units were clustered using UCLUST. To maintain analytical consistency, the reads were normalized to 9,260 per sample. Beta diversity was visualized using principal coordinate analysis (PCoA) based on generalized UniFrac distances at the species level, which accounts for both abundant and rare lineages. To balance the contributions of common and rare taxa, the weighting parameter α was set to 0.5 [27]. The relative abundances of bacterial taxa were calculated for comparison between groups, and the selected taxa were graphically presented using GraphPad Prism (ver. 8.01).

### Microbial functional profiling and Linear Discriminant analysis

Functional metagenomic potential was predicted via Phylogenetic Investigation of Communities by Reconstruction of Unobserved States (PICRUSt) based on the Kyoto Encyclopedia of Genes and Genomes (KEGG) orthology. MinPath was used for pathway reconstruction [25,26]. Taxonomic biomarkers were identified using the Linear Discriminant Analysis (LDA) effect size (LEfSe). Differences among groups were initially evaluated using the Kruskal–Wallis test (*p <* 0.05), and taxa showing consistent variation across subgroups were further assessed using pairwise Wilcoxon tests. Features with a logarithmic LDA score > 2.0 were considered significant. Selected taxa and pathway results were visualized using GraphPad Prism and RStudio.

### Statistical analysis

All data are presented as mean ± SEM. Statistical analyses were conducted using the SPSS software (version 20.0; IBM Corp., Armonk, NY, USA). The beta-diversity of the microbial communities was analyzed separately. All statistical analyses were performed separately within the HK-BBH024 and L-BBH024 experimental rounds. No statistical comparisons were performed between the two experimental rounds. Continuous and categorical variables were compared between groups (no-stress control, WAS, and WAS with BBH024 treatment) using the Kruskal–Wallis test for continuous variables. Pairwise comparisons were performed using the Mann–Whitney U test when appropriate. Correlations between mRNA expression and pathological phenotypes, including average FPO and mast cell count, were assessed using Spearman’s rank correlation with complete pairwise observations. Differences and correlations were considered statistically significant at *p <* 0.05. No global adjustment for multiple comparisons was applied to the pairwise comparisons or correlation analyses unless otherwise specified. Accordingly, nominal *p* values from these analyses were interpreted cautiously, particularly for exploratory microbiota and correlation analyses. For beta-diversity comparisons, both *p* and *q* values provided by the PERMANOVA analysis are reported.

### Accession number of data

All datasets generated in this study have been deposited in the National Center for Biotechnology Information Sequence Read Archive (NCBI SRA) under accession numbers (no. PRJNA1461423)

## Results

### Effects of two BBH024 preparations on rWAS-induced FPO and colonic mast cell infiltration

Because the HK-BBH024 and L-BBH024 experiments were conducted independently, all treatment effects described below refer to comparisons within the corresponding experimental round. During the experimental period, no significant differences in body weight were observed among groups of the same sex, although a distinct weight difference was observed between male and female rats (S2 Fig). Following 10 days of WAS, the mean FPO during the 1-hour sessions significantly increased in both sexes compared to the CON group (male WAS groups, *p <* 0.001; female WAS groups, *p <* 0.001; Fig 1A, 1B). Within the HK-BBH024 experiment, HK-BBH024 administration significantly reduced FPO in both male and female rats compared with their corresponding WAS groups (male WAS + HK-BBH024, *p =* 0.006; female WAS + HK-BBH024, *p =* 0.017; Fig 1A). Similarly, within the independently conducted L-BBH024 experiment, L-BBH024 administration significantly reduced FPO in both sexes compared with their corresponding WAS groups (male WAS + L-BBH024, *p =* 0.002; female WAS + L-BBH024, *p <* 0.001; Fig 1B). These treatment-associated reductions in FPO were also observed in the raw, unadjusted data in both sexes (S1 Fig). When comparing the daily cumulative FPO, all WAS groups showed a significant increase starting from day 1 (*p <* 0.05, Fig 1C-1F). In the HK-BBH024 experiment, the male treatment group showed a decrease in FPO compared to the WAS group; the difference was not statistically significant (Fig 1C). However, the female HK-BBH024 group exhibited a significant reduction in FPO from days 4–6 (*p <* 0.05; Fig 1D). Similarly, in the L-BBH024 trial, while the male treatment group showed a non-significant decreasing trend, the female L-BBH024 group significantly decreased (*p <* 0.05) in FPO from day 3 to the end of the experiment (day 10) (Fig 1E, 1F). In the HK-BBH024 experiment, mast cell counts increased in the WAS groups and showed a downward trend following probiotic administration in both sexes; however, these changes were not statistically significant (Fig 1G). In the L-BBH024 experiment, the WAS groups of both sexes showed a significant increase in mast cell counts compared with the CON group (male WAS, *p =* 0.026; female WAS, *p =* 0.001; Fig 1H). While the male L-BBH024 group showed a non-significant reduction, the female L-BBH024 group exhibited a borderline decrease in mast cell counts compared to the WAS group (female WAS + L-BBH024, *p =* 0.050; Fig 1H).

**Fig 1:**
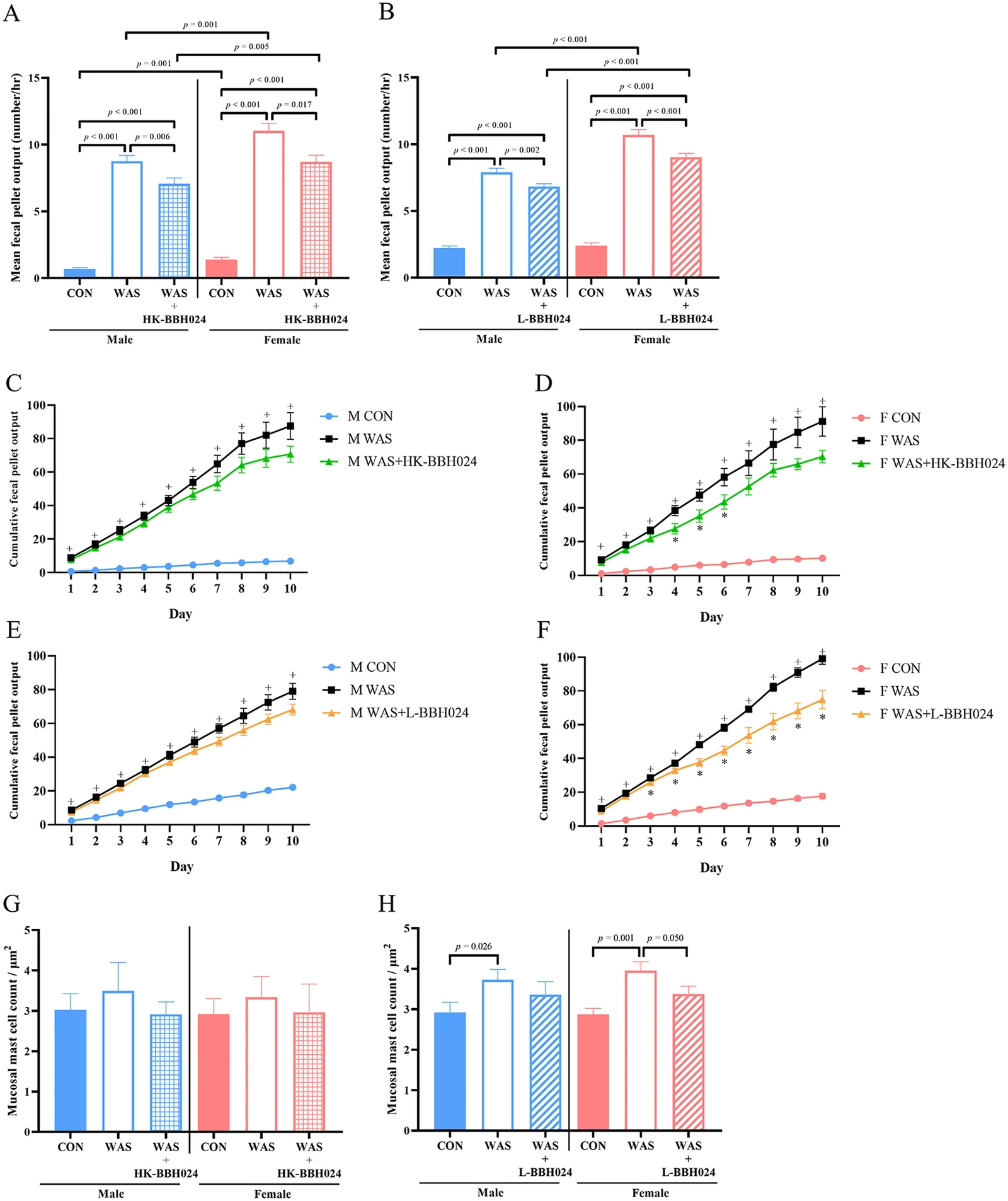
Fecal pellet output and mast cell counts in the repeated water avoidance stress experiment. Average fecal pellet output (FPO) during 1-h water avoidance stress (WAS) sessions for 10 consecutive days in the HK-BBH024 and L-BBH024 experiments, respectively (A, B). Female FPO values were normalized based on the mean body weight ratio between male and female rats. Daily cumulative FPO during the experimental period is shown in (C–F). Symbols indicate statistically significant differences between groups: + , CON vs. WAS; *, WAS vs. BBH024 treatment; *p <* 0.05. Quantification of mast cell infiltration in the proximal colonic mucosa using toluidine blue-stained sections is shown in (G, H). Data are presented as mean ± SEM. Statistical significance was determined using the Mann–Whitney U-test for pairwise comparisons, and exact p-values are shown in the graphs. n = 6 per group. CON, control; WAS, water avoidance stress; HK-BBH024, heat-killed *R. faecis*; L-BBH024, viable cell-containing *R. faecis* preparation; FPO, fecal pellet output.

### Effects of HK-BBH024 and L-BBH024 on WAS-associated colonic protease gene expression in male and female rats

To further evaluate whether *R. faecis* modulates stress-associated molecular changes in the colon, mRNA expression levels of serine protease-related genes and *IL-1b* were analyzed in the colonic mucosa. In the HK-BBH024 experiment, the expression levels of *Prss1, Prss2*, and *Prss3* did not differ significantly among the CON, WAS, and HK-BBH024-treated groups in male and female rats (Fig 2A–2C). Similarly, *IL-1b* expression tended to increase in the WAS groups compared with the CON groups in both sexes, but this increase was not statistically significant, and HK-BBH024 administration did not significantly alter *IL-1b* expression (Fig 2D). In contrast, the L-BBH024 experiment revealed a sex-specific regulation of protease-related gene expression. Under WAS conditions, the expression levels of *Prss1* (*p =* 0.002), *Prss2* (*p =* 0.006), and *Prss3* (*p =* 0.027) were significantly higher in female rats than in male rats (Fig 2E–2G). In female rats, *Prss1* (*p =* 0.007) and *Prss2* (*p =* 0.001) expression levels were significantly higher in the WAS group than those in the CON group, and these increases were significantly attenuated by L-BBH024 administration (*Prss1* in female WAS + L-BBH024, *p =* 0.010; *Prss2* in female WAS + L-BBH024, *p =* 0.010; Fig 2E, 2F). No significant changes in *Prss1* or *Prss2* expression were observed in the male groups (Fig 2E, 2F). Conversely, *Prss3* expression was significantly higher in male rats following WAS exposure than in the CON group (*p =* 0.024). This increase was reduced by L-BBH024 treatment (*p =* 0.021), whereas no significant differences in *Prss3* expression were detected in female rats (Fig 2G). *IL-1b* expression did not show significant differences among groups in either sex in the L-BBH024 experiment (Fig 2H). In the HK-BBH024 experiment, no significant treatment-associated changes in the colonic mucosal expression of *Prss1*, *Prss2*, *Prss3*, or *IL-1b* were observed. In the independently conducted L-BBH024 experiment, L-BBH024 treatment significantly attenuated WAS-associated changes in *Prss1* and *Prss2* expression in females and *Prss3* expression in males.

**Fig 2:**
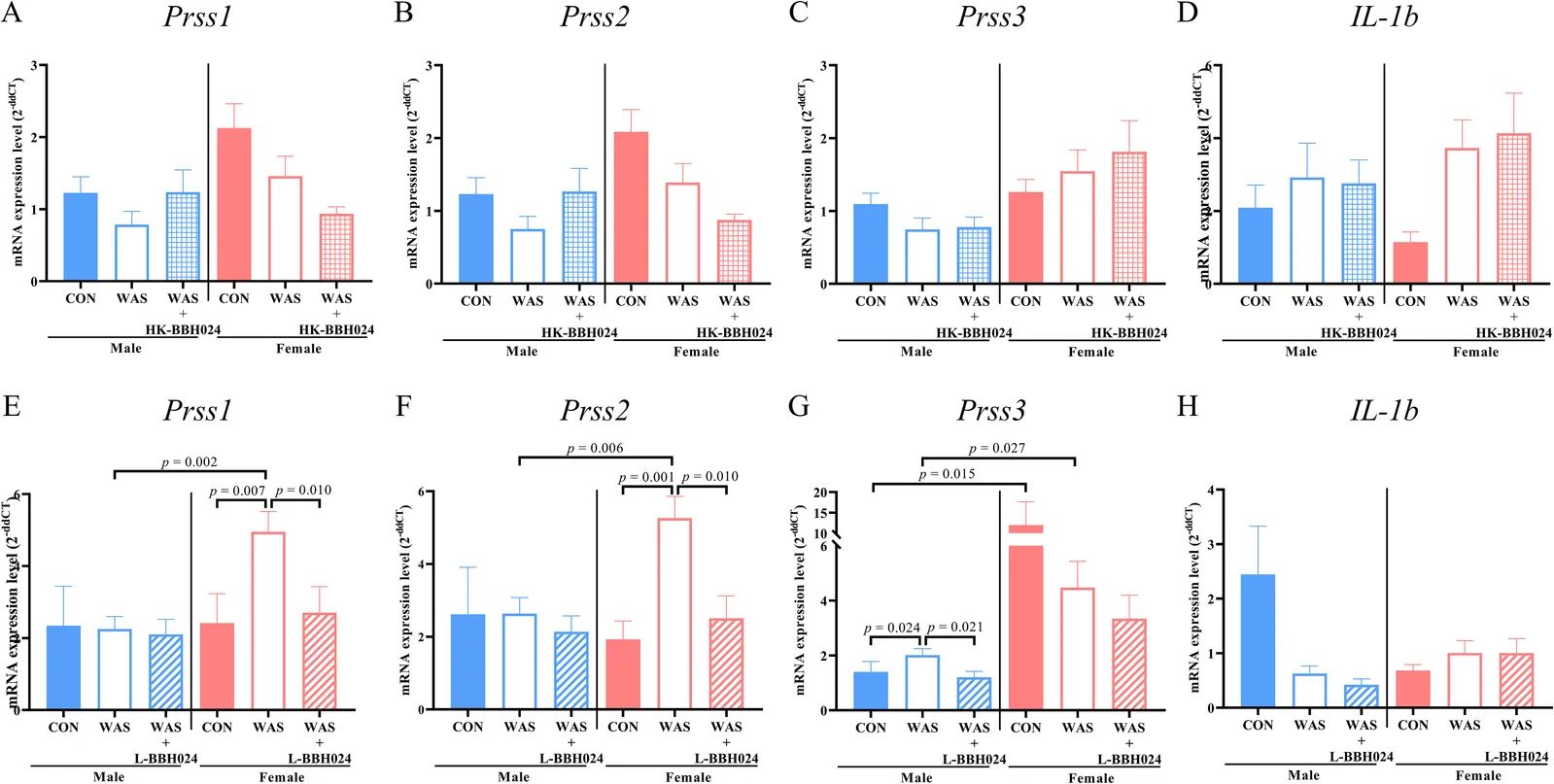
Colonic mucosal mRNA expression of protease-related genes and *IL-1b* following WAS and *R. faecis* treatment. The mRNA expression levels of *Prss1*, *Prss2*, *Prss3*, and *IL-1b* were analyzed in colonic mucosa. Expression levels in the HK-BBH024 experiment are shown in A–D, and those in the L-BBH024 experiment are shown in E–H. *Prss1* expression is shown in A and E, *Prss2* expression in B and F, *Prss3* expression in C and G, and *IL-1B* expression in D and H. Data are presented as mean ± SEM. Statistical significance was determined using the Mann–Whitney U-test for pairwise comparisons. n = 6 per group. CON, control; WAS, water avoidance stress; HK-BBH024, heat-killed *R. faecis*; L-BBH024, viable cell-containing *R. faecis* preparation*.*

### Sex differences in response to WAS and *R. faecis* BBH024 treatment

Sex differences were observed in the WAS-induced responses and the effects of *R. faecis* treatment. In both the independently conducted HK-BBH024 and L-BBH024 experiments, WAS-induced FPO was significantly higher in female rats than in male rats (*p <* 0.001; Fig 1A, 1B), indicating a greater functional response to stress in females. Different treatment-associated FPO patterns were observed in male and female rats. In the daily cumulative FPO analysis, male rats showed no significant reduction in the HK- and L-BBH024 groups compared to the WAS group (Fig 1C, 1E). In contrast, female rats showed a significant reduction in FPO levels following the administration of both the preparations (Fig 1D, 1F). A similar numerical pattern was observed for the colonic mast cell counts. Following L-BBH024 administration, mast cell infiltration showed a borderline decrease in female rats (*p =* 0.050), whereas the reduction in male rats was not statistically significant (Fig 1H). In the L-BBH024 experiment, different gene-specific response patterns were observed in male and female rats. *Prss1* and *Prss2* expression levels were significantly increased by WAS and were significantly reduced by L-BBH024 treatment in female rats, whereas no significant changes were observed in male rats (Fig 2E, 2F). In contrast, *Prss3* expression was significantly increased by WAS treatment and reduced by L-BBH024 treatment in male rats, whereas no significant changes were observed in female rats (Fig 2G). These results show that different response patterns were observed in male and female rats, with more evident FPO reductions in females and different patterns of protease-related gene responses in the L-BBH024 experiment.

### Correlations among FPO, mast cell counts, and mucosal gene expression

Correlation analyses were performed to assess the relationships among average FPO values, colonic mast cell counts, and mucosal mRNA expression levels. In the HK-BBH024 experiment, *IL-1b* expression was positively correlated with both average FPO (*p =* 0.001) and mast cell counts (*p =* 0.045) (Fig 3A, 3B). In the L-BBH024 experiment, mast cell counts were positively correlated with the average FPO (*p =* 0.020, Fig 3C). Average FPO was also positively correlated with *Prss1* and *Prss2* expression levels (*p =* 0.001, Fig 3D, 3E), and mast cell counts were positively correlated with *Prss1* expression (*p =* 0.022, Fig 3F). No significant correlations were detected in the other comparisons (S2 Fig).

**Fig 3:**
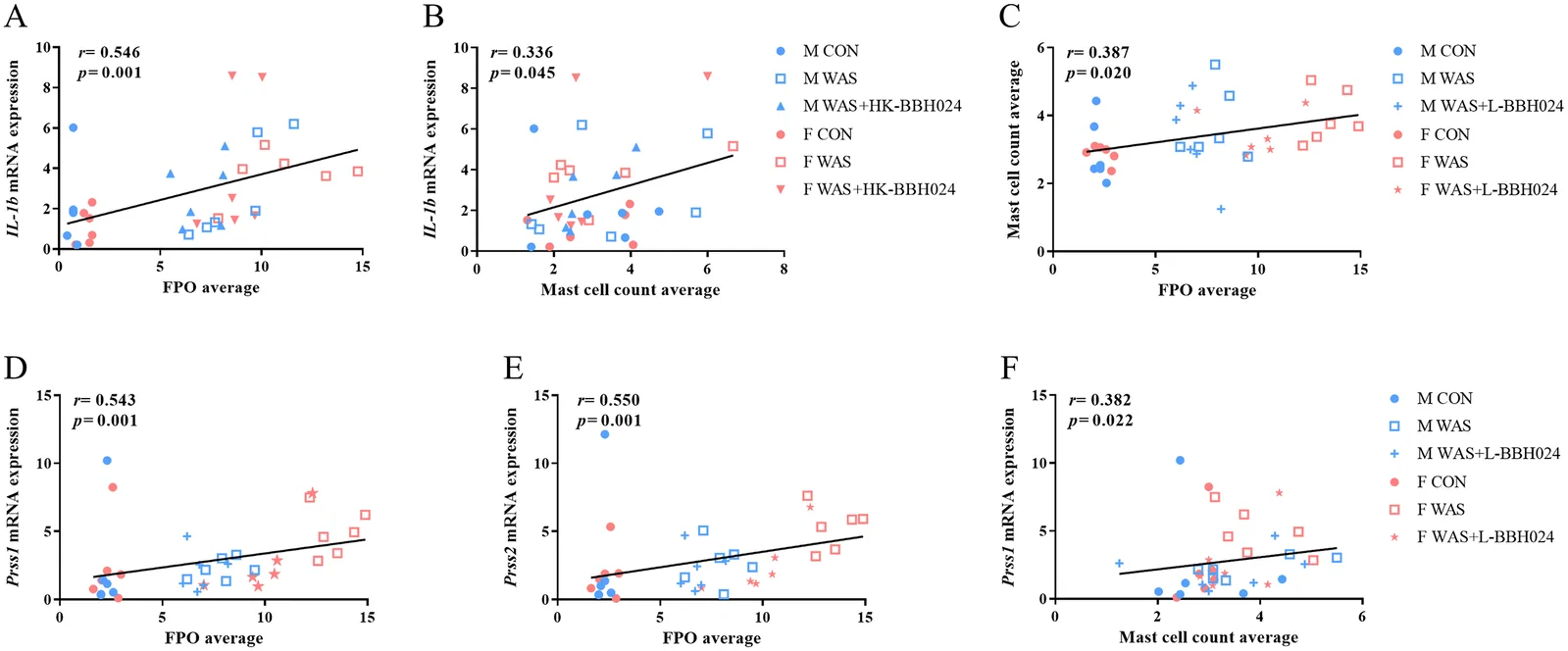
Correlations among fecal pellet output, mast cell counts, and colonic mucosal mRNA expression. Correlations in the HK-BBH024 experiment are shown in A and B, and correlations in the L-BBH024 experiment are shown in C–F. Spearman’s correlation analysis was used, and correlation coefficients (r) and p-values are shown in each graph. n = 6 per group. FPO, fecal pellet output; CON, control; WAS, water avoidance stress; HK-BBH024, heat-killed *R. faecis*; L-BBH024, viable cell-containing *R. faecis* preparation*.*

### Effects of WAS and *R. faecis* treatment on fecal microbiota composition

Changes in fecal microbiota induced by WAS and *R. faecis* administration were analyzed using 16S rRNA gene sequencing. At the phylum level, the HK-BBH024 experiment showed a tendency toward increased Bacteroidetes and decreased Firmicutes abundance in male rats following WAS exposure; however, these changes were not restored by HK-BBH024 administration (Fig 4A). In the L-BBH024 experiment, no significant changes in phylum abundance were observed following WAS or L-BBH024 treatment in either sex (Fig 4B). Consistently, the Firmicutes/Bacteroidetes ratio did not differ significantly among groups in either the HK- or L-BBH024 experiments, and no significant changes were observed in other phyla (S3 Fig).

**Fig 4:**
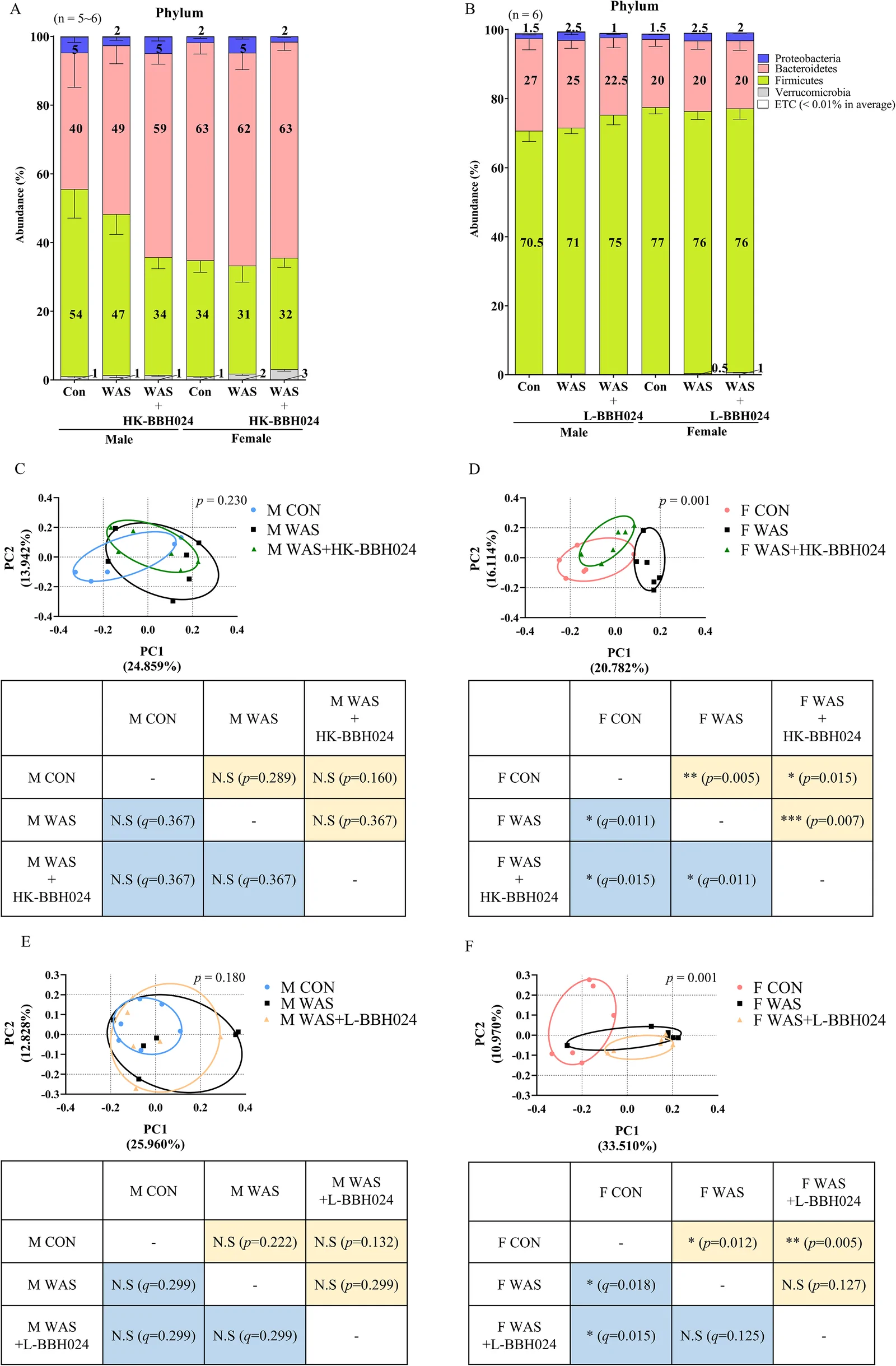
Fecal microbiota phylum level composition and beta-diversity following WAS and *R. faecis* treatment. Fecal microbiota composition was analyzed by 16S rRNA gene sequencing. Phylum-level relative abundance in the HK-BBH024 and L-BBH024 experiments is shown in A and B, respectively. Principal coordinate analysis (PCoA) plots of beta-diversity are shown for male and female rats in the HK-BBH024 experiment (C, D) and in the L-BBH024 experiment (E, F). Pairwise comparisons of microbial community composition between groups were performed using the PERMANOVA method on the EzBioCloud platform, and corresponding *p*- and *q*-values are summarized in the tables below each plot (all pairwise comparisons shown above the graphs). PC1 explains 24.859% of the variance, PC2 explains 13.942% of the variance for (C) male HK-BBH024 experiment rats and PC1 explains 20.782% of the variance, PC2 explains 16.114% of the variance for (D) female HK-BBH024 experiment rats. PC1 explains 25.960% of the variance, PC2 explains 12.828% of the variance for (E) male L-BBH024 experiment rats and PC1 explains 33.510% of the variance, PC2 explains 10.970% of the variance for (F) female L-BBH024 experiment rats. n = 6 per group, except for the male CON group in the HK-BBH024 experiment used for NGS analysis, where one sample was excluded during quality control (n = 5). M, male; F, female; CON, control; WAS, water avoidance stress; HK-BBH024, heat-killed *R. faecis*; L-BBH024, viable cell-containing *R. faecis* preparation.

Beta diversity was assessed using PCoA analysis. In the HK-BBH024 experiment, male rats showed no significant differences in the microbial community structure among the CON, WAS, and HK-BBH024 groups (Fig 4C). In contrast, significant differences in female rats were noted between the CON and WAS (*p =* 0.005, *q =* 0.011), WAS and HK-BBH024(*p =* 0.015, *q =* 0.015), and CON and HK-BBH024 groups (*p =* 0.007, *q =* 0.011) (Fig 4D). In the L-BBH024 experiment, no significant differences in beta diversity were detected among the male groups (Fig 4E). In female rats, the microbial community structure differed significantly between the CON and WAS groups (*p =* 0.012, *q =* 0.018), whereas no significant differences were observed between the WAS and L-BBH024 groups (Fig 4F). Alpha diversity indices further revealed sex differences in microbial diversity. In the HK-BBH024 experiment, ACE (*p =* 0.018) and Chao1 (*p =* 0.045) indices were significantly higher in female CON rats than in male CON rats (S4 Fig). In the L-BBH024 experiment, female WAS rats showed significantly lower NPShannon (*p =* 0.037) and Shannon (*p =* 0.037) indices and a significantly higher Simpson index (*p =* 0.007) than female CON rats; however, these indices did not differ significantly between the WAS and L-BBH024 groups (S4 Fig). In addition, female CON rats showed significantly higher NPShannon (*p =* 0.004) and Shannon (*p =* 0.004) indices, and a significantly lower Simpson index (*p =* 0.003) than male CON rats in the L-BBH024 experiment (S4 Fig). Overall, significant microbiota changes were detected mainly in beta diversity and were more evident in female than in male rats, whereas phylum-level abundance and alpha diversity showed limited changes after WAS or BBH024 administration.

### Effects of HK-BBH024 and L-BBH024 on WAS-associated changes in selected bacterial taxa

To further characterize the taxonomic changes associated with the WAS and BBH024 treatments, the microbial abundance at the genus and species levels was analyzed. At the genus level, the HK-BBH024 experiment revealed several significant changes in female rats. The relative abundance of *Roseburia* tended to increase in the WAS group compared to that in the CON group, although this change was not statistically significant, a significant decrease was observed following HK-BBH024 administration compared to that in the WAS group (*p =* 0.025; Fig 5A). *Bacteroides* abundance was significantly higher in the female WAS group than in the female CON group (*p =* 0.007); however, its reduction after HK-BBH024 treatment was not statistically significant (Fig 5B). *Blautia* abundance was also significantly increased by WAS in female rats compared to that in the CON group (*p =* 0.004), and was significantly decreased following HK-BBH024 administration compared to that in the WAS group (*p =* 0.008; Fig 5C). In contrast, no significant changes in *Lactobacillus* abundance were observed in the HK-BBH024 experiment (Fig 5D). In the L-BBH024 experiment, the abundance of *Lactobacillus* was significantly lower in the WAS group than that in the CON group in both male and female rats (*p =* 0.037 for both sexes). Although *Lactobacillus* abundance increased following L-BBH024 administration in both sexes, these changes were not statistically significant (Fig 5H). No significant changes were observed in the relative abundance of *Roseburia*, *Bacteroides*, or *Blautia* in the L-BBH024 experiment (Fig 5E–5G).

**Fig 5:**
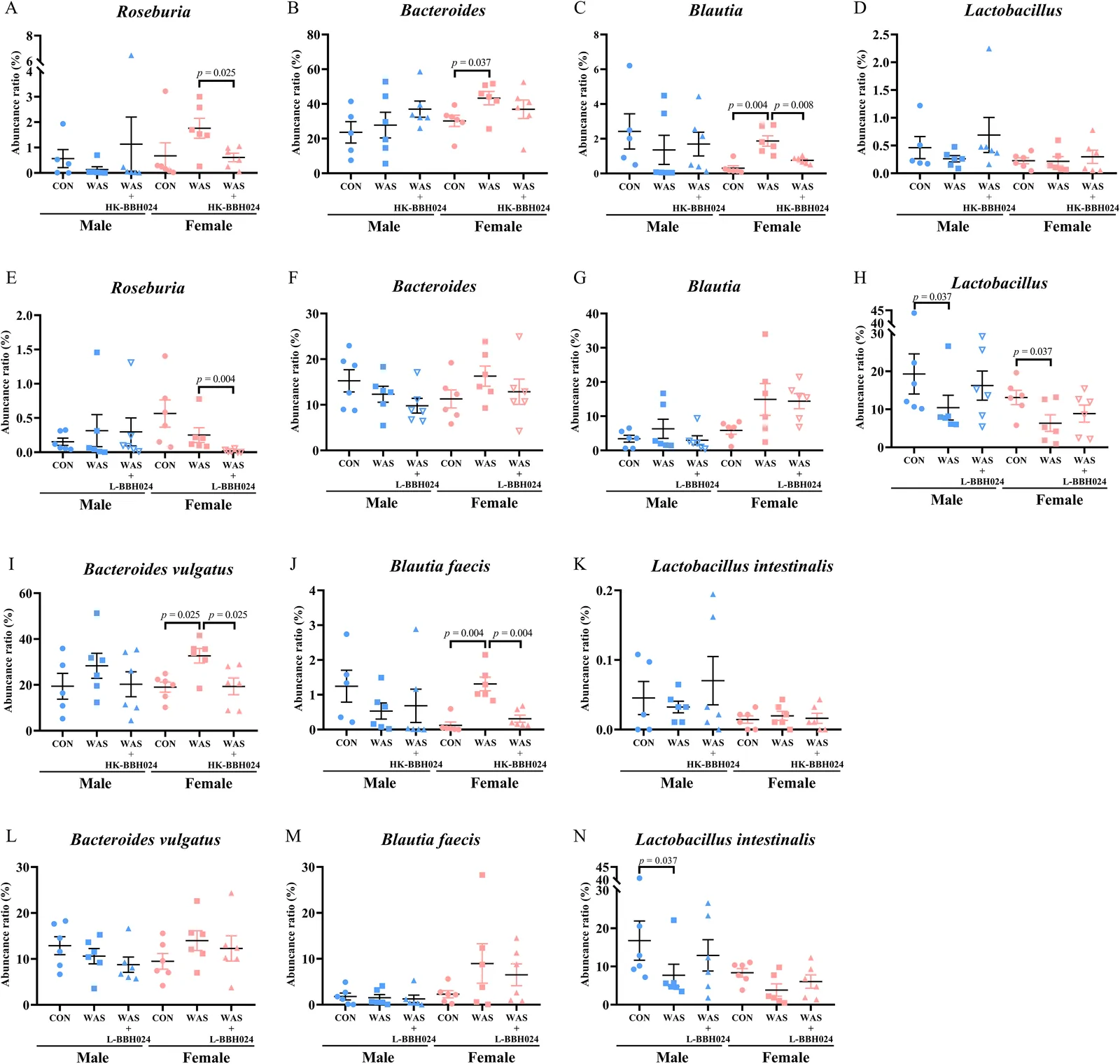
Genus- and species-level fecal microbiota changes after WAS and *R. faecis* BBH024 treatment. Relative abundance ratio of selected bacterial genera were analyzed in fecal samples from the HK-BBH024 and L-BBH024 experiments. In the HK-BBH024 experiment, the abundance ratio of *Roseburia*, *Bacteroides*, *Blautia* and *Lactobacillus* were compared among CON, WAS, and WAS + HK-BBH024 groups (A–D). In the L-BBH024 experiment, the abundance ratio of *Roseburia*, *Bacteroides*, *Blautia* and *Lactobacillus* were compared among CON, WAS, and WAS + L-BBH024 groups (E–H). Data are presented as mean ± SEM. Statistical significance was determined using the Mann–Whitney U-test for pairwise comparisons. n = 6 per group, except for the male CON group in the HK-BBH024 experiment used for NGS analysis, where one sample was excluded during quality control (n = 5). CON, control; WAS, water avoidance stress; HK-BBH024, heat-killed *R. faecis*; L-BBH024, viable cell-containing *R. faecis* preparation*.*

At the species level, the HK-BBH024 experiment showed significant changes, particularly in female rats. The relative abundance of *Bacteroides vulgatus* was significantly higher in the female WAS group than in the female CON group (*p =* 0.023), and was significantly decreased following HK-BBH024 administration compared to the WAS group (*p =* 0.023; Fig 5I). Similarly, *Blautia faecis* abundance was significantly increased by WAS in female rats compared to that in the CON group (*p =* 0.004) and significantly decreased after HK-BBH024 treatment compared to that in the WAS group (*p =* 0.004; Fig 5J). No significant changes were observed in male rats in the HK-BBH024 experiment, and *Lactobacillus intestinalis* abundance did not differ significantly among the groups of either sex (Fig 5I–5K). In the L-BBH024 experiment, *Lactobacillus intestinalis* abundance was significantly lower in male WAS rats than in male CON rats (*p =* 0.037) and tended to increase after L-BBH024 treatment, although this change was not statistically significant (Fig 5N). No significant changes in *L. intestinalis* abundance were observed in the female rats. In addition, *B. vulgatus* and *B. faecis* abundance did not differ significantly among groups of either sex in the L-BBH024 experiment (Fig 5L, 5M).

### LEfSe analysis revealed sex different microbial biomarkers altered by WAS and *R. faecis* treatment

LEfSe analysis was performed to identify the differentially enriched species-level taxa among the CON, WAS, and BBH024-treated groups. Only taxa showing significant enrichment are presented in Fig 6, and all taxa described below showed statistical significance at *p <* 0.05. In the HK-BBH024 experiment, male CON rats showed a higher enrichment of commensal taxa, including *Collinsella aerofaciens* group, *Dorea formicigenerans*, and *Coprococcus catus*, than the WAS group (Fig 6A). Compared to WAS, HK-BBH024 treatment was associated with enrichment of the *Lactobacillus gasseri* group. Notably, *Blautia glucerasea* was enriched in both the CON and HK-BBH024 groups compared to that in the WAS group (Fig 6A). In female rats from the HK-BBH024 experiment, excluding unclassified taxa, *B. glucerasea* was enriched compared to the WAS group (Fig 6B). Compared with the female CON group, the WAS group showed enrichment of commensal taxa, including the *Anaerostipes hadrus* group, *Blautia luti*, *Blautia wexlerae*, and *Parabacteroides goldsteinii*. In addition, *D. formicigenerans* was enriched in the WAS group compared to the HK-BBH024 group. When compared with both the CON and HK-BBH024 groups, the WAS group showed overlapping enrichment of *Dorea massiliensis*, *C. catus*, *Fusicatenibacter saccharivorans*, and *Blautia faecis*, together with the potentially pathobiont-associated taxon *B. vulgatus* (Fig 6B). In the L-BBH024 experiment, male CON rats showed enrichment of commensal taxa, including *Bifidobacterium pseudolongum* and *Lactobacillus intestinalis*, compared to the WAS group (Fig 6C). Conversely, the WAS group showed an enrichment of potentially pathobiont-associated taxa, including *Robinsoniella peoriensis* and *Escherichia coli*, compared to the CON group. When comparing male WAS and L-BBH024 groups, *Clostridium celatum* was enriched in the WAS group (Fig 6C). In female rats in the L-BBH024 experiment, commensal taxa, including *F. saccharivorans*, *L. gasseri* group, *Roseburia cecicola* group, and *Flintibacter butyricus* group, were more enriched in the CON group than in the WAS group (Fig 6D). Compared to WAS, L-BBH024 treatment was associated with the enrichment of *B. glucerasea*. In contrast, compared to the CON group, the WAS group showed an enrichment of commensal taxa, including *Clostridium asparagiforme*, *Longicatena caecimuris*, *Ruthenibacterium lactatiformans*, and *D. formicigenerans*, together with the context-dependent taxon *Collinsella aerofaciens*. Compared with the L-BBH024 group, the WAS group showed enrichment of *C. catus* and *Romboutsia timonensis*, together with the potential pathobiont-associated taxon *Bacteroides caccae*. Notably, in comparison to WAS, *B. pseudolongum* was enriched in both the CON and L-BBH024 groups. On the other hand, *P. goldsteinii* was enriched in the WAS group compared with both groups (Fig 6D).

**Fig 6:**
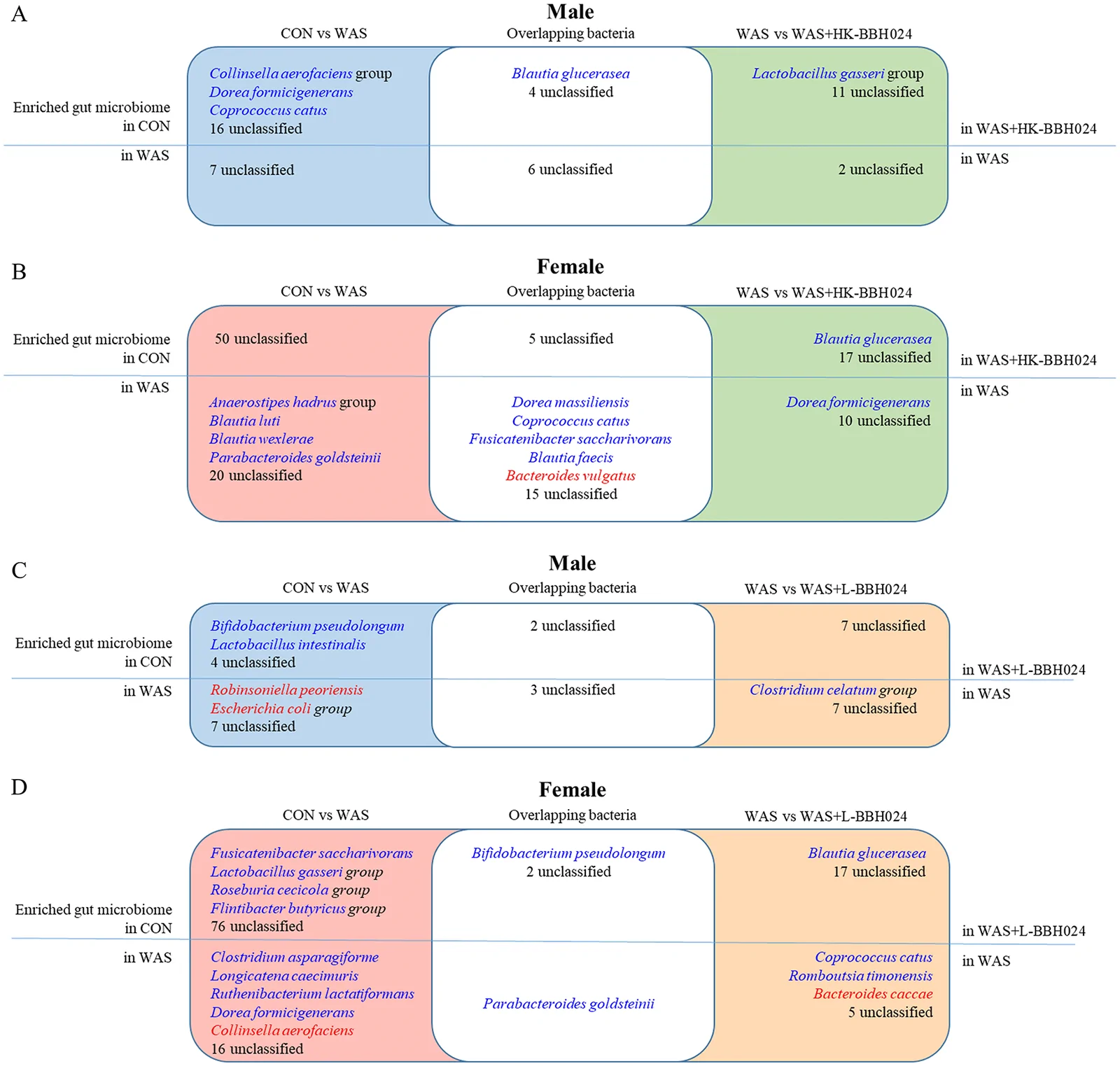
Species-level microbial biomarkers identified by LEfSe after WAS and *R. faecis* BBH024 treatment. Species-level taxa differentially enriched among CON, WAS, and *R. faecis* treated groups were identified using linear discriminant analysis effect size (LEfSe) analysis. Venn diagrams summarize significantly enriched taxa and overlapping taxa identified in pairwise comparisons among groups. Species-level microbial biomarkers in male (A) and female (B) rats from the HK-BBH024 experiment. Species-level microbial biomarkers in male (C) and female (D) rats from the L-BBH024 experiment. Taxa names shown in blue indicate commensal taxa, whereas names shown in red indicate potentially pathobiont associated taxa. Only taxa with significant enrichment are shown (*p <* 0.05). n = 6 per group, except for the male CON group in the HK-BBH024 experiment used for NGS analysis, where one sample was excluded during quality control (n = 5). CON, control; WAS, water avoidance stress; HK-BBH024, heat-killed *R. faecis*; L-BBH024, viable cell-containing *R. faecis* preparation*.*

### Predictive functional profiling revealed sex different alterations in microbial metabolic potential after WAS and BBH024 treatment

Predictive functional profiling was performed using KEGG pathway-based analysis with MinPath to assess differences in microbial functional potential among groups. Functional LEfSe analysis was performed across the CON, WAS, and BBH024-treated groups, and the revised presentation was restricted to pathways classified under KEGG Metabolism. The mean predicted functional abundances of the three groups were visualized using heatmaps. In the heat maps, a deeper red indicates a higher predicted functional abundance, whereas a deeper blue indicates a lower predicted functional abundance. In the HK-BBH024 experiment, tryptophan metabolism (ko00380) differed significantly among the three male groups, with its mean predicted abundance progressively increasing across the CON, WAS, and HK-BBH024 groups (Fig 7A). In females, valine, leucine and isoleucine degradation (ko00280), lipopolysaccharide biosynthesis (ko00540), and lipoic acid metabolism (ko00785) differed significantly among the three groups. The mean predicted abundances of valine, leucine and isoleucine degradation and lipopolysaccharide biosynthesis were lower in the WAS group than in the CON group and higher in the HK-BBH024 group than in the WAS group, whereas lipoic acid metabolism showed the opposite pattern (Fig 7B). In the L-BBH024 experiment, no significantly altered metabolic pathways were identified in male rats. In females, valine, leucine and isoleucine biosynthesis (ko00290), galactose metabolism (ko00052), secondary bile acid biosynthesis (ko00121), pyrimidine metabolism (ko00240), vitamin B6 metabolism (ko00750), and peptidoglycan biosynthesis (ko00550) differed significantly among the three groups. The mean predicted abundances of these pathways were higher in the WAS group than in the CON group and lower in the L-BBH024 group than in the WAS group.

**Fig 7:**
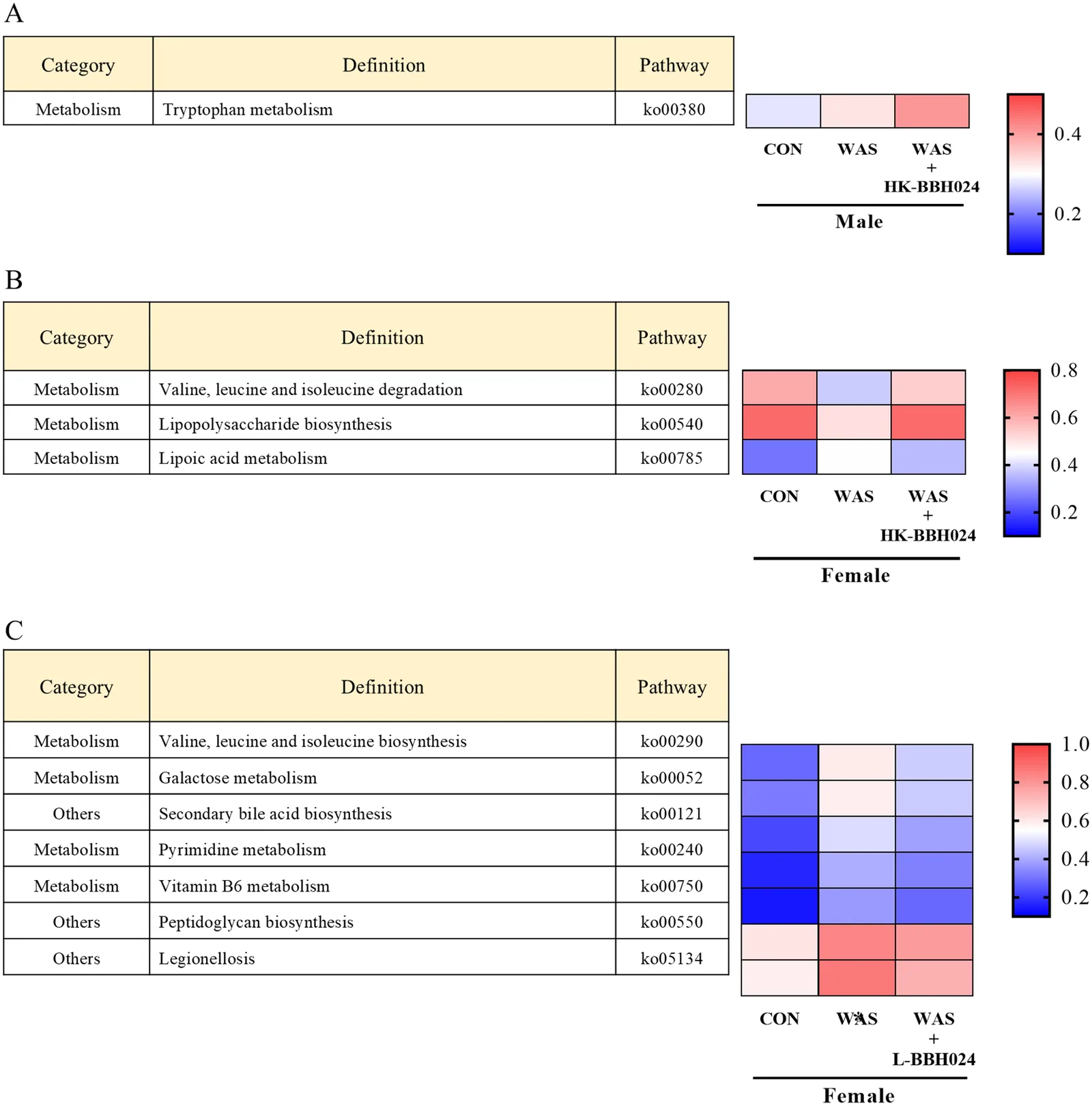
Sex-specific predicted microbial functional changes after WAS and *R. faecis* BBH024 treatment. Predicted microbial functional profiles were analyzed using KEGG pathway-based functional prediction with MinPath and functional LEfSe analysis and visualized as heatmaps. Only pathways classified under KEGG Metabolism that differed significantly among the CON, WAS, and BBH024-treated groups are shown (*p <* 0.05). Heatmap colors represent the mean predicted functional abundance of each group. Deeper red indicates higher predicted functional abundance, whereas deeper blue indicates lower predicted functional abundance. In the HK-BBH024 experiment, altered predicted metabolic pathways are shown for males (A) and females (B). In the L-BBH024 experiment, altered predicted metabolic pathways are shown for females (C). No significantly altered metabolic pathways were identified in male rats in the L-BBH024 experiment. n = 6 per group, except for the male CON group in the HK-BBH024 experiment used for NGS analysis, where one sample was excluded during quality control (n = 5). CON, control; WAS, water avoidance stress; HK-BBH024, heat-killed *R. faecis*; L-BBH024, viable cell-containing *R. faecis* preparation.

## Discussion

Within their respective experimental rounds, HK-BBH024 and L-BBH024 were each associated with reductions in WAS-induced FPO. In the L-BBH024 experiment, additional treatment-associated changes were observed in colonic mucosal mast cell infiltration and *Prss* related gene expression, with different patterns observed in male and female rats. The increases in FPO were similar to those observed in our previous study, in which two *R. faecis* strains, BBH024 and R22-12–24, reduced rWAS-induced FPO and colonic mast cell infiltration [6]. In that study, BBH024 and R22-12–24 reduced FPO elevation following rWAS exposure, and colonic mast cell infiltration was attenuated under the selected conditions. In the R22-12–24 experiment, these effects were also accompanied by changes in the fecal microbiota and predicted microbial function [6]. The present study extended these findings by evaluating HK-BBH024 and L-BBH024 in separate experimental rounds: both preparations were produced at industrial scale from the same BBH024 strain. The use of these preparations reflected practical issues encountered during industrial-scale production of *R. faecis*, wherein maintaining high viability is technically challenging because this bacterium is an obligate anaerobe [28]. Accordingly, we evaluated an industrially processed heat-killed BBH024 preparation and compared it with a viable cell containing BBH024 preparation in separate experimental rounds. Because HK-BBH024 and L-BBH024 were evaluated in independently conducted experimental rounds with separate CON and WAS groups, the present study was not designed to directly determine the relative efficacy of the two preparations or the specific contribution of bacterial viability. Rather, the findings characterize the treatment-associated effects observed with each preparation within its respective experimental setting.

FPO increased by rWAS can be interpreted as a functional indicator of stress-induced colonic hypermotility and IBS-related defecation abnormalities [6,7,29]. In the present study, HK-BBH024 and L-BBH024 each reduced the average FPO relative to the corresponding WAS group within their respective experimental rounds (Fig 1A, 1B). The reduction observed following HK-BBH024 administration suggests that non-viable *R. faecis*-derived bacterial components may contribute to modulation of stress-induced defecation responses.. However, analysis of daily cumulative FPO showed that the treatment-associated reduction was more evident in female rats. In the HK-BBH024 experiment, female rats showed significant reductions form days 4–6 (Fig 1D). In the L-BBH024 experiment, female rats showed significant reductions were observed form day 3 through day 10 (Fig 1F). Overall, treatment-associated reductions in FPO were observed in both experimental rounds, with more evident responses in female rats. Because the HK-BBH024 and L-BBH024 experiments were conducted independently, the differences in the duration of these responses cannot be interpreted as evidence of greater efficacy of either preparation. The female-predominant response observed in our study may be related to sex differences in susceptibility to stress-induced colonic responses in the rWAS model. A recent study also reported higher WAS-induced FPO levels and significant increases in mast cells in female rats, although the treatment effects were more pronounced in male rats, suggesting that sex-dependent responses may vary depending on the intervention or microbial strain [30]. Such variation may partly reflect differences in the host pathways engaged by individual microbial interventions, as male and female immune responses can differ in cellular signaling and immunometabolic states, some of which are influenced by sex hormones [31]. Accordingly, intervention-specific effects on different host pathways may contribute to the sex-stratified response patterns observed across studies, although this possibility requires direct mechanistic and interaction analyses. These findings suggest that host sex may be associated with differences in stress responses and treatment-associated outcomes in WAS-induced IBS models.

In addition to functional bowel responses, mast cell activation and *Prss*-related serine protease signaling appear to contribute to stress-related mucosal microinflammation in IBS by affecting epithelial permeability, sensory nerve activation, barrier dysfunction, and visceral hypersensitivity [32]. Therefore, the combined assessment of FPO, colonic mucosal mast cell infiltration, and *Prss* expression allowed us to evaluate the effects of BBH024 in terms of functional, histological, and molecular perspectives. In the HK-BBH024 experiment, treatment was associated with reduced FPO, whereas significant treatment associated changes in mast cell counts and *Prss*-related gene expression were not detected. In the independently conducted L-BBH024 experiment, a borderline treatment-associated decrease in mast cell infiltration was observed in female rats, whereas significant reductions were observed in *Prss1* and *Prss2* expression in females and *Prss3* expression in males (Fig 1G, 1H; Fig 2E-2G). These findings show treatment-associated, sex- and gene-specific differences in *Prss* mRNA expression following L-BBH024 administration, accompanied by a borderline decrease in mast cell infiltration in females. The positive correlations between FPO and mast cell count and the expression of *Prss1* and *Prss2* were consistent with an association between WAS-induced defecation and these mucosal responses (Fig 3C-3F). However, because *Prss* expression was assessed only at the mRNA level, this study did not directly demonstrate changes in protease activity. Therefore, these findings should be interpreted as treatment-associated changes in *Prss* mRNA expression rather than evidence of altered protease activity or a protease-mediated mechanism.

Fecal microbiota analysis showed that changes induced by WAS and BBH024 treatment were not broadly reflected at the phylum-level composition or alpha-diversity level but were instead observed in beta-diversity, selected taxa, LEfSe-derived biomarkers, and predicted functional profiles, with different patterns between male and female rats. In particular, alterations in the microbial community structure were more evident in female rats than in male rats, which was partly consistent with the treatment-associated FPO reductions and the borderline decrease in mast cell infiltration observed in female rats. Different within-experiment patterns in selected fecal bacterial taxa were observed across the HK-BBH024 and L-BBH024 experimental rounds. *Blautia* abundance was significantly increased by WAS treatment and decreased after HK-BBH024 treatment (Fig 5C). At the species level, *Blautia faecis* and *B*. *vulgatus* showed similar patterns, with a significant increase in female WAS rats, and a significant decrease following HK-BBH024 administration (Fig 5I, 5J). *B. vulgatus* is a common gut commensal species that exhibits pathological features under inflammatory or dysbiotic conditions. Therefore, the parallel reduction in *B. faecis* and *B. vulgatus* after HK-BBH024 treatment may reflect the partial modulation of WAS-associated dysbiosis in female rats. L-BBH024 did not induce clear restorative changes in the selected genus- or species-level taxa, although *Lactobacillus* and *L. intestinalis* showed non-significant increases after treatment under some conditions (Fig 5H, 5N). Collectively, the microbiota data suggest that rWAS-associated changes and BBH024 treatment-associated responses varied according to host sex and across the independently conducted experimental rounds. The present design does not permit these differences to be attributed specifically to the type of BBH024 preparation.

LEfSe analysis further revealed different microbial biomarker patterns in male and female rats across the independently conducted experimental rounds. Rather than indicating a uniform shift from commensal taxa to potentially pathobiont-associated taxa, LEfSe results suggested complex taxonomic rearrangements that varied according to sex and experimental round. In the HK-BBH024 experiment, several commensal taxa, including *B. glucerasea* were enriched in male CON and HK-BBH024 treated groups compared to the WAS group (Fig 6A), whereas female WAS rats showed enrichment of *B. vulgatus*, consistent with the genus- and species-level abundance results (Fig 6B). In the L-BBH024 experiment, *B. pseudolongum* was enriched in the female CON and L-BBH024-treated groups (Fig 6D). Predictive functional profiling also revealed sex specific patterns in predicted KEGG pathways within the respective experimental rounds. In female rats from the L-BBH024 experiment, predicted functional pathways related to valine, leucine and isoleucine biosynthesis, secondary bile acid biosynthesis, galactose metabolism, pyrimidine metabolism, vitamin B6 metabolism, and peptidoglycan biosynthesis were increased by WAS and decreased after L-BBH024 treatment (Fig 7C). Impaired bile acid regulation and excessive fecal bile acid excretion have been associated with IBS-D [33,34]. As the gut microbiota plays a key role in bile acid metabolism [35], the observed changes in the predicted secondary bile acid biosynthesis may indicate differences in predicted microbial functional potential associated with WAS and L-BBH024 treatment. However, PICRUSt-based KEGG analysis is a predictive approach based on 16S rRNA sequencing data, and does not directly demonstrate microbial gene expression, metabolite production, or fecal bile acid profiles. Therefore, these functional profiling results should be interpreted as hypothesis-generating findings requiring further validation using approaches such as shotgun metagenomics, metabolomics, and fecal bile acid analyses.

This study had several limitations. First, HK-BBH024 and L-BBH024 were evaluated in separate experimental rounds conducted at different times, and each round included independent CON and WAS groups. Therefore, the study was not designed for a direct head-to-head comparison of the two preparations, and differences observed across the two experimental rounds should not be interpreted as evidence that either preparation was more effective. Second, L-BBH024 contained only a limited proportion of viable cells, and the HK-BBH024 and L-BBH024 preparations differed in total cell density despite being administered at the same preparation mass. Therefore, the present study cannot distinguish the contribution of bacterial viability from potential effects related to total cell dose. Third, we analyzed only the fecal microbiota, and mucosa-associated or luminal microbiota were not directly assessed. In addition, intestinal colonization or persistence of the administered *R. faecis* BBH024 was not directly assessed. Therefore, the observed microbiota alterations cannot be attributed to stable colonization of BBH024 and may also reflect transient or indirect effects of the administered preparation on the gut microbial community. PICRUSt-predicted functional pathways, including secondary bile acid biosynthesis, were not experimentally validated and should be confirmed in future studies using metagenomic, metabolomic, or direct bile acid analyses. In addition, because the present study was conducted in rats and microbiota profiling was based on 16S rRNA sequencing, caution is required when extrapolating these findings to the human gut microbiota. Other components of the gut microbial ecosystem and interspecies interactions that may influence *Roseburia* abundance or host responses were also not comprehensively assessed. Fourth, *Prss1–3* expression was measured only at the mRNA level, without direct assessment of fecal protease or trypsin-like activity. Fifth, visceral hypersensitivity, a core feature of IBS and the rWAS model, was not directly assessed. Therefore, the FPO findings should be interpreted as changes in stress-induced defecation rather than evidence that BBH024 alleviated overall IBS-like responses. Finally, the relatively small sample size, limited the statistical power and lack of direct assessment of sex hormone levels, estrous cycle, or stress hormone responses limit the mechanistic interpretation of the observed sex differences. Because hormonal status can influence immune responses, gut microbial composition, and stress responsiveness, the biological mechanisms underlying the observed differences between male and female rats remain speculative and require further investigation. In addition, physiological stress markers such as corticosterone, adrenocorticotropic hormone (ACTH), and corticotropin-releasing hormone (CRH) were not measured; therefore, the extent of stress-axis activation and its potential modulation by BBH024 could not be directly assessed. Given that formal sex-by-treatment interaction analyses were not performed, the observed differences between male and female rats should be interpreted as sex-stratified patterns rather than statistically confirmed sex-dependent treatment effects. In addition, because a comprehensive multiple-testing correction was not applied across all pairwise, correlation, and microbiota analyses, the possibility of false-positive findings cannot be excluded. Therefore, particularly the correlation and exploratory microbiota findings should be interpreted cautiously and require confirmation in independent studies.

In conclusion, within their respective experimental rounds, both HK-BBH024 and L-BBH024 reduced rWAS-induced FPO, with treatment-associated reductions being more evident in female rats. HK-BBH024 was associated with female-specific modulation of selected fecal taxa, including *B. faecis* and *B. vulgatus*. In the L-BBH024 experiment, treatment was associated with reductions in cumulative FPO and *Prss1* and *Prss2* expression, as well as a borderline decrease in mast cell infiltration, in females. L-BBH024 was also associated with differences in PICRUSt-predicted microbial functional pathways, including predicted secondary bile acid biosynthesis, within the L-BBH024 experiment. Given the technical difficulty of maintaining high viability of *R. faecis* during industrial-scale production, the FPO-lowering effect of HK-BBH024 suggests that heat-killed preparations may still offer a practical postbiotic-like strategy. However, future direct head-to-head studies using preparations with substantially higher viability and appropriately matched total cell doses under identical experimental conditions are required to determine the specific contribution of viable BBH024 cells.

## Supporting information

S1 Fig Raw, unnormalized fecal pellet output in male and female rats. Raw FPO data from the (A) HK-BBH024 and (B) L-BBH024 experiments are presented without adjustment for body weight. Data are presented as mean ± SEM. Statistical significance was determined using the Mann–Whitney U-test for pairwise comparisons, and exact p-values are shown in the graphs. n = 6 per group. CON, control; WAS, water avoidance stress; HK-BBH024, heat-killed *R. faecis*; L-BBH024, viable cell-containing *R. faecis* preparation; FPO, fecal pellet output.
(TIF)

S2 Fig Body weight changes and additional correlation analyses. Body weight changes during the experimental period are shown in A and B. Additional correlation analyses among average fecal pellet output (FPO), mast cell counts, and colonic mucosal mRNA expression levels are shown in C–N. Spearman’s correlation analysis was used, and correlation coefficients (r) and p-values are shown in each graph. Data are presented as mean ± SEM where applicable. n = 6 per group. FPO, fecal pellet output; CON, control; WAS, water avoidance stress; HK-BBH024, heat-killed *R. faecis*; L-BBH024, viable cell-containing *R. faecis* preparation*.*
(TIF)

S3 Fig Phylum level relative abundance ratio following WAS and *R. faecis* treatment. Phylum level relative abundance and Firmicutes/Bacteroidetes ratio (F/B ratio) were analyzed in fecal samples from the HK-BBH024 and L-BBH024 experiments. The Firmicutes/Bacteroidetes ratio is shown in A and D, Proteobacteria in B and E, and Verrucomicrobia in C and F. Data are presented as mean ± SEM. Statistical significance was determined using the Mann–Whitney U-test for pairwise comparisons. n = 6 per group, except for the male CON group in the HK-BBH024 experiment used for NGS analysis, where one sample was excluded during quality control (n = 5). F/B, Firmicutes/Bacteroidetes; CON, control; WAS, water avoidance stress; HK-BBH024, heat-killed *R. faecis*; L-BBH024, viable cell-containing *R. faecis* preparation*.*
(TIF)

S4 Fig Alpha-diversity indices of fecal microbiota following WAS and *R. faecis* treatment. Alpha-diversity of fecal microbiota was assessed using multiple diversity indices in the HK-BBH024 and L-BBH024 experiments. Alpha-diversity indices in the HK-BBH024 experiment are shown in A–I, and those in the L-BBH024 in J–R. Data are presented as mean ± SEM. Statistical significance was determined using the Mann–Whitney U-test for pairwise comparisons, and exact p-values are depicted. n = 6 per group, except for the male CON group in the HK-BBH024 experiment used for NGS analysis, where one sample was excluded during quality control (n = 5). CON, control; WAS, water avoidance stress; HK-BBH024, heat-killed *R. faecis*; L-BBH024, viable cell-containing *R. faecis* preparation*.*
(TIF)

S1 Data Underlying numerical data for body weight changes, fecal pellet output, RT-qPCR, taxonomic LEfSe, and MinPath analyses. (XLSX)

S2 Data Underlying microbiota abundance and diversity data from the phylum to species levels, including alpha- and beta-diversity analyses. (XLSX)
